# An Unusual Presentation of a Myocardial Crypt in Hypertrophic Cardiomyopathy

**DOI:** 10.1155/2014/737052

**Published:** 2014-11-12

**Authors:** Danny A. J. P. van de Sande, Jan Hoogsteen, Luc J. H. J. Theunissen

**Affiliations:** Department of Cardiology, Máxima Medical Center, De Run 4600, 5504 DB Veldhoven, The Netherlands

## Abstract

Hypertrophic cardiomyopathy (HCM) is a common inherited cardiovascular disease with prevalence of 0.2% in the population. More than 1000 mutations in more than 10 genes encoding for proteins of the cardiac sarcomere have been identified. Cardiac magnetic resonance imaging (CMR) is used to characterize left ventricular morphology with great precision in patients with HCM and it identifies unique structural abnormalities in patients with HCM. We present a case of a 56-year-old man who had positive family history of HCM who was a carrier of the genetic MYH-7 2770 G > C, exon 23 mutation. Transthoracic echocardiography showed thickening of the interventricular septum (16 mm) and in particular the basal septum. CMR confirmed the diagnosis of HCM in the anteroseptal myocardium with a thickness of 23 mm and also revealed large and deep myocardial crypts in the anterior wall. These myocardial crypts are rarely found in the so-called genotype positive and phenotype positive patients, as in our case. Also the crypts in this case are deeper and wider than those reported in other cases. So in conclusion, this case reveals an uncommon finding of a myocardial crypt at an unusual myocardial site with the unusual morphology in a patient with genotypic and phenotypic expression of hypertrophic cardiomyopathy.

## 1. Case Presentation

**Figure 1:**
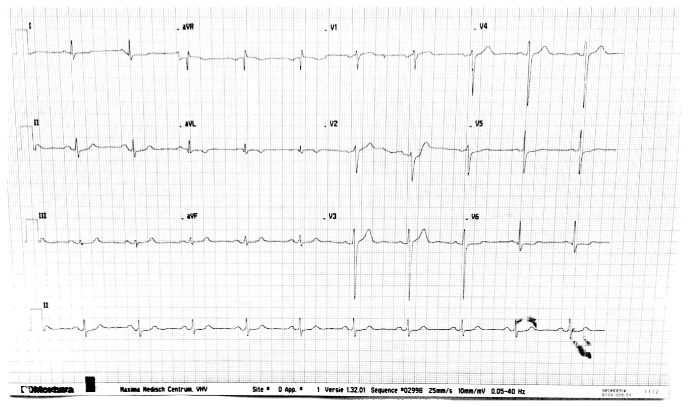
The 12-lead electrocardiogram of the patient presented in the case. The ECG revealed sinus rhythm with normal conduction and with modest repolarization abnormalities in leads aVL and V6.

**Figure 2:**
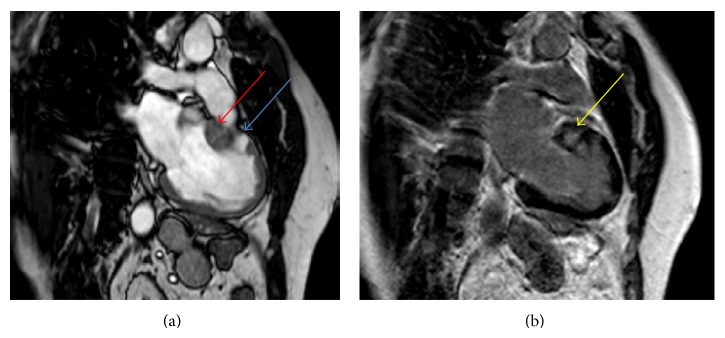
Cardiac magnetic resonance 2-chamber cine view imaging of the patient presented in the case. (a) CMR 2-chamber cine image with the myocardial crypt (blue arrow) in the anteroseptal segment on the left and hypertrophy (red arrow) of the basal interventricular septum. (b) CMR 2-chamber image delayed enhancement with mid wall fibrosis in the basal anterior segment (yellow arrow).

**Figure 3:**
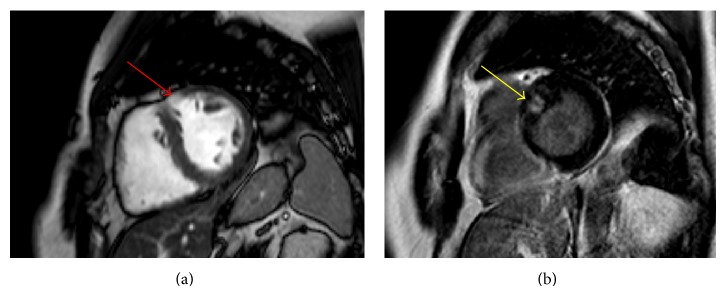
Cardiac magnetic resonance short-axis imaging of the patient presented in the case. (a) CMR short-axis cine image with the myocardial crypt (red arrow) in the anteroseptal segment on the left. (b) CMR short-axis image delayed enhancement in the anteroseptal segment with mid wall fibrosis (yellow arrow). Images (a) and (b) are not oriented in the same slice position due to image quality.

A 56-year-old male was referred to the Department of Cardiology of the Maxima Medical Centre for the evaluation of possible hypertrophic cardiomyopathy (HCM). The patient had no complaints of dyspnea, shortness of breath, chest pain, fatigue, sensation of palpitations, dizziness, or edema. No cardiovascular risk factors like the use of tobacco, hypertension, or diabetes mellitus were present and no medication was used. The patient had a positive family history for HCM and the patient was carrier of the genetic MYH-7 2770 G > C, exon 23 mutation. This is a common mutation and together with the MYBPC3 mutation is accountable for approximately 70% in patients with genotyped HCM [1]. Physical examination revealed no abnormalities. The electrocardiogram revealed sinus rhythm with modest repolarization abnormalities in leads aVL and V6 (Figure 1). Subsequent transthoracic echocardiography showed slight enlargement of the left atrium. Left and right ventricular functions were within normal range with an ejection fraction of 70%. The interventricular septum, especially the basal part, was thickened with a thickness of 16 millimeters. There was no evidence of contraction abnormalities or outflow obstruction. For further assessment and to confirm the suspected HCM, cardiac magnetic resonance (CMR) was performed. The CMR confirmed the thickened anteroseptal myocardium with a thickness of 23 mm and focal delayed enhancement within the basal anteroseptal segment with mid wall fibrosis was also present (Figures 2 and 3). CMR also revealed large myocardial crypts in the anterior wall (Figures 2 and 3). Additional finding on CMR was marked enlargement of both of the atria. The diagnosis of HCM was confirmed in a genotype and phenotype positive patient. The patient remained asymptomatic to date. No myocardial biopsy was performed.

## 2. Discussion

Hypertrophic cardiomyopathy is a common inherited cardiovascular disease with prevalence of 0.2% in the population. More than 1000 mutations in more than 10 genes encoding for proteins of the cardiac sarcomere have been identified [1]. A thickened (hypertrophied) nondilated left ventricle (LV) characterizes HCM in the absence or without evidence of a cardiac or systemic disease, which is able to produce such a grade of hypertrophy [1, 2]. This thickening can be detected by 2D echocardiography and CMR provides an advanced imaging tool to characterize the phenotypic expression of HCM [3]. Recently, CMR studies identified a structural abnormality in patients with HCM, called the myocardial crypt. Germans et al. [4] revealed that in more than 80% of genotype positive (carrier of one of the proven genetic mutations for HCM) and phenotype negative (without LV hypertrophy) patients this abnormal structure of the myocardium was found. This abnormality was not found in any of the healthy volunteers. However, the current case is different, as this patient had profound phenotypic abnormalities, matching HCM. This patient was the so-called genotype positive and phenotype positive patient. Maron et al. [3] investigated the prevalence and clinical profile of myocardial crypts in HCM. That study revealed that, in accordance with Germans et al. [4], myocardial crypts were found in more than 60% of genotype positive and phenotype negative patients. However, like in this case, myocardial crypts were present in only 4% of the phenotypic affected patients [3]. All these crypts were confined to the basal half of the LV and most commonly were located in the posterior septum (70%) [3]. Also, the location of the crypt in our case was not reported in patients with any form of the MYH-7 mutation [3]. In 90% delayed enhancement was found, which is a common finding in HCM [5] but not in the same segment as the crypts were situated. In this case, the myocardial crypt was found in the anterior septum, reported only in 10% by Maron et al. [3] and delayed enhancement was present in the same segment as the myocardial crypt. Also, the crypt in this case was deep and wide and it differs considerably in morphology from the relative narrow crypts reported by Maron et al. [3]. Such a deep and wide crypt is reported in only one other case report and was seen in the basal ventricular septum [6]. However, the clinical significance of this morphology of the crypt in patients with HCM is unknown. In conclusion, this case reveals an uncommon finding of a myocardial crypt at an unusual myocardial site with the unusual morphology in a patient with genotypic and phenotypic expression of hypertrophic cardiomyopathy.

## References

[1] Maron B. J., Maron M. S. (2013). Hypertrophic cardiomyopathy. *The Lancet*.

[2] Cannavale A., Ordovás K. G., Rame E. J., Higgins C. B. (2010). Hypertrophic cardiomyopathy with restrictive phenotype and myocardial crypts. *Journal of Thoracic Imaging*.

[3] Maron M. S., Rowin E. J., Lin D., Appelbaum E., Chan R. H., Gibson C. M., Lesser J. R., Lindberg J., Haas T. S., Udelson J. E., Manning W. J., Maron B. J. (2012). Prevalence and clinical profile of myocardial crypts in hypertrophic cardiomyopathy. *Circulation: Cardiovascular Imaging*.

[4] Germans T., Wilde A. A. M., Dijkmans P. A., Chai W., Kamp O., Pinto Y. M., van Rossum A. C. (2006). Structural abnormalities of the inferoseptal left ventricular wall detected by cardiac magnetic resonance imaging in carriers of hypertrophic cardiomyopathy mutations. *Journal of the American College of Cardiology*.

[5] Hansen M. W., Merchant N. (2007). MRI of hypertrophic cardiomyopathy: part I, MRI appearances. *The American Journal of Roentgenology*.

[6] Maron B. J., Lindberg J., Lesser J. R. (2010). Ventricular septal crypt in hypertrophic cardiomyopathy. *European Heart Journal*.

